# MANIPULATING THE ENDORSEMENT OF PRESCRIPTIVE VIEWS OF AGING—SPECIFICITY AND INTERNALIZATION

**DOI:** 10.1093/geroni/igad104.0895

**Published:** 2023-12-21

**Authors:** Maria Wirth, M Clara P de Paula Couto, Klaus Rothermund

**Affiliations:** Friedrich Schiller University Jena, Jena, Thuringen, Germany; Friedrich Schiller University Jena, Jena, Thuringen, Germany; Friedrich Schiller University Jena, Jena, Thuringen, Germany

## Abstract

Prescriptive views of aging are beliefs about how older adults should be and behave. The two most prominent views entail that older adults should remain active and contribute to society (active aging) and that they should withdraw from important positions (altruistic disengagement). Prescriptive views of aging set societal standards for age (in)appropriate behaviour and many older adults internalize these norms so much that they appear completely natural to them. This study investigated the malleability of endorsing prescriptive norms using social consensus information: 367 participants (50–87 years) were asked to indicate whether the general public agreed or disagreed with altruistic disengagement or active aging in the health or social domain. Subsequently, participants were presented with (fictitious) social consensus feedback which was either in line with participants’ estimation of social consensus or not (random assignment). Our results indicate that presenting social consensus feedback affected the endorsement of prescriptive views of aging. Learning that a majority of others supported (dis)agreement with a prescriptive age norm led to (lower) higher endorsement of this norm. This effect was specific to the norm and domain for which consensus feedback was presented. Thus, activation and disengagement are represented as separate norms in a domain-specific fashion. Endorsing prescriptions for older adults, in general, led participants to also more strongly endorse this norm for themselves. Our findings attest to the complexity and specificity of prescriptive views of aging and offer important insights for designing interventions.

